# Expression of Connexins 37, 43 and 45 in Developing Human Spinal Cord and Ganglia

**DOI:** 10.3390/ijms21249356

**Published:** 2020-12-08

**Authors:** Marija Jurić, Julia Zeitler, Katarina Vukojević, Ivana Bočina, Maximilian Grobe, Genia Kretzschmar, Mirna Saraga-Babić, Natalija Filipović

**Affiliations:** 1 Department of Anatomy, Histology and Embryology, Laboratory for Neurocardiology, University of Split School of Medicine, Šoltanska 2, 21000 Split, Croatia; maarjur@gmail.com (M.J.); zeitlerjulia@gmx.de (J.Z.); kvukojev@gmail.com (K.V.); maxgrobe@gmx.de (M.G.); genia.kretzschmar@yahoo.com (G.K.); 2 Department of Anatomy, Histology and Embryology, Laboratory for Early Human Development, University of Split School of Medicine, Šoltanska 2, 21000 Split, Croatia; msb@mefst.hr; 3 Faculty of Science, University of Split, Ruđera Boškovića 33, 21000 Split, Croatia; bocina@pmfst.hr

**Keywords:** human embryo, spinal cord, dorsal root ganglia, connexin

## Abstract

Direct intercellular communication via gap junctions has an important role in the development of the nervous system, ranging from cell migration and neuronal differentiation to the formation of neuronal activity patterns. This study characterized and compared the specific spatio-temporal expression patterns of connexins (Cxs) 37, 43 and 45 during early human developmental stages (since the 5th until the 10th developmental week) in the spinal cord (SC) and dorsal root ganglia (DRG) using double immunofluorescence and transmission electron microscopy. We found the expression of all three investigated Cxs during early human development in all the areas of interest, in the SC, DRG, developing paravertebral ganglia of the sympathetic trunk, notochord and all three meningeal layers, with predominant expression of Cx37. Comparing the expression of different Cxs between distinct developmental periods, we did not find significant differences. Specific spatio-temporal pattern of Cxs expression might reflect their relevance in the development of all areas of interest via cellular interconnectivity and synchronization during the late embryonic and early fetal period of human development.

## 1. Introduction

The neural circuits encompassed in the spinal cord (SC) serve as efferent pathways, being responsible for the somatic motor and visceral functions, but also as afferent pathways transmitting important incoming data for the body like temperature and proprioception [1]. The complex structure of the human central nervous system (CNS) begins to develop in the third week of embryogenesis. The ectoderm starts to form a thick plate of the neuroepithelium, called the neural plate, in the mid-dorsal region anterior to the primitive node. This plate is the source of neurons and glial cells of the CNS. The neural folds are created by elevating the lateral ends of the neural plate in the process named primary neurulation. After growing, the neural folds approach each other in the cranial and caudal direction and form the neural tube. The wider cranial part and the narrower caudal part of the tube are precursors of the brain and the SC, respectively. Secondary neurulation describes the formation of the neural tube, which arises from a solid cord of cells that submerge into the embryo, and then subsequently undergo cavitation to form a hollow tube. Additionally, these cells give rise to neurons, neuroglia, and ependymal cells. Neural crest cells migrate from the dorsal most region of the neural tube to form different ganglia located on the dorsal part of the SC, paravertebral and prevertebral region (sympathetic neurons), and into the effector organs (parasympathetic neurons).

The SC derives from the neural tube [2,3]. The structure of the neural tube is divided into three layers: the inner layer (INL) called a ventricular layer, lining the cavity of the neural tube, the future central canal, the intermediate layer referred to as the mantle layer, and the outer layer named the marginal layer (marginal zone–mz) [4]. The wall of the central canal becomes thickened by the formation of neuroblasts in the mantle layer. Due to the location of cell bodies, this part of the SC will later form the grey matter. The axons of these neurons will grow into the marginal layer, later referred to as white matter [4]. A pair of grooves, called sulcus limitans, appear in the cross-section at approximately the midpoints, along the lateral margin of the neural canal during the proliferation of neuroblasts. Cells migrating dorsally from the sulcus limitans form the alar plate (AP), whereas cells migrating ventrally form the basal plate (BP) [4]. At the dorsal position, the roof plate (RP), and at the ventral aspect, the floor plate (FP), is formed. The BPs contain the motor area in the developing SC, while the APs contain the sensory areas [4]. The future intermediate horn originates from the neurons in an intermediate position to the sulcus limitans and will fulfill autonomic functions. Opposite to the APs and BPs, the RP and FP do not contain neuroblasts but nerve fibers [5]. Dorsal root ganglia (DRG), contained in the dorsal root, are part of the peripheral nervous system and relay peripheral afferent stimuli through the processes of their pseudounipolar neurons into the CNS. The branch of the axon entering the SC either directly synapses with neurons in the posterior gray column or continues to ascend [5,6]. The embryonic origin of the DRG is the neural crest cells [7].

The different functions of the SC are mirrored anatomically for those aforementioned functions (afferent and efferent) allocated to distinct regions of the SC during embryonic development [2,5]. The development of different subgroups of cells in the evolving SC happens along the spatial axes of the embryo and is furthermore governed by temporal changes in the expression of morphogens [2,8,9]. The cell differentiation along the rostral-caudal axis is determined by concentration gradients of rostralizing retinoic acid and caudalizing fibroblast growth factor [2,10]. The dorsal-ventral patterning of the SC is governed by concentration gradients of secreted Sonic hedgehog (Shh) protein and bone morphogenetic proteins (BMPs) [5,8]. Ectoderm overlying the neural tube is the initial tissue secreting BMPs, which then leads to the secretion of BMPs by the RP itself. BMPs and Wnts are the factors governing the dorsalizing specialization of the neural tube cells [5,8]. The ventral signal, Shh, inducing neural cell differentiation of FP cells, motor neurons, and ventral interneurons is provided by the notochord and then by the FP itself [2,5,11]. The concentration gradients of morphogenetic signals exert their function through regulation of transcription factors which then initiate the specialization into ventral and dorsal cell subtypes [5].

Besides the morphogenetic signal, numerous studies have shown that direct intercellular communication via gap junctions has an important role in the development of the nervous system [12,13,14,15,16,17,18]. Gap junctions are integral parts of the cellular membrane, providing a way of electrical and metabolic communication between cells through the rapid exchange of ions, metabolites, and second messengers [19,20]. This communication also plays an important role in embryogenesis, due to the coordination of migrating cell masses and developmental regulation [21]. Each gap junction is formed via the pairing of two hemichannels called connexons, each consisting of six connexins (Cx) proteins being located on adjacent cells. While each specific Cx can form its own specific gap junction, in many cases, gap junctions consist of more than one Cx isoform, creating heteromeric connexons with more than two different Cxs or heterotypic gap junctions with different connexons [22,23]. Cxs are named according to their specific molecular mass, with the most common having a mass of 43 kDa and therefore being called Cx43. Until now, 21 human genes and 20 mouse genes for Cx expression have been identified [24]. While some are expressed in various types of tissues, most Cxs have a tissue-specific expression pattern, allowing a classification based on the organs they are expressed in [25]. In the SC, Cxs are expressed in glial and neuronal cells. As part of the gap junctions, they have a major role in intercommunication between glia and neurons as they are essential for ion transfer, propagation of the inflammatory response, control of glial proliferation, myelination, and differentiation of neuronal stem cells [26,27]. In addition, presynaptic and postsynaptic neurons can be bridged by gap junctions, with the possible spreading of the electrical potential in both directions [28].

Despite the extensive research of Cx distribution and function in the CNS development, there is a lack of information about the expression of different types of Cxs in the embryonal development of the SC, especially in the human embryo. Hence, the aim of this study was to characterize the expression of different types of Cxs in SC and DRG during early human developmental stages, which could imply their role in embryonal/early fetal SC development.

## 2. Results

**Figure 1:**
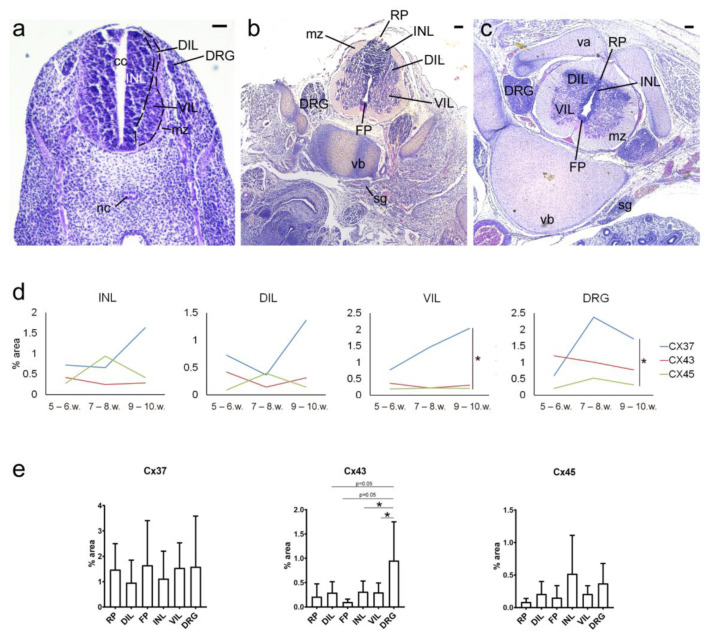
Hematoxylin-eosin staining of developing human spinal cord (SC) and ganglia and analysis of different connexins (Cxs) expression. Photomicrographs of sections through the thoracic segment of the human SC in (**a**) 5 weeks (objective magnification—10×; scale bar = 80 µm), (**b**) 6.5 weeks, and (**c**) 10 week-old human conceptuses (both objective magnification—4×; scale bars = 200 µm). DRG—dorsal root ganglion; INL—inner layer; DIL—dorsal intermediate zone; VIL—ventral intermediate zone; mz—marginal zone; cc—central canal; RP—roof plate; FP—floor plate; nc—notochord; sg—sympathetic ganglion; vb—vertebral body; va—vertebral arch. (**d**) Timeline of expression of different Cxs in different areas of the SC and DRG. (**e**) Expression of Cx37, Cx43, and Cx45 in different areas of the SC (for all samples). * *p* < 0.05 between indicated Cxs/areas.

We studied the expression of Cx37, Cx43, and Cx45 in different parts of the SC during the embryonal and early fetal period. Next, areas of interest were included: INL, dorsal part of the intermediate layer—DIL (corresponding to the AP), and ventral part of the IL—VIL (corresponding to the BP) of the intermediate zone, RP, and FP, as well as DRG (Figure 1).

**Figure 2:**
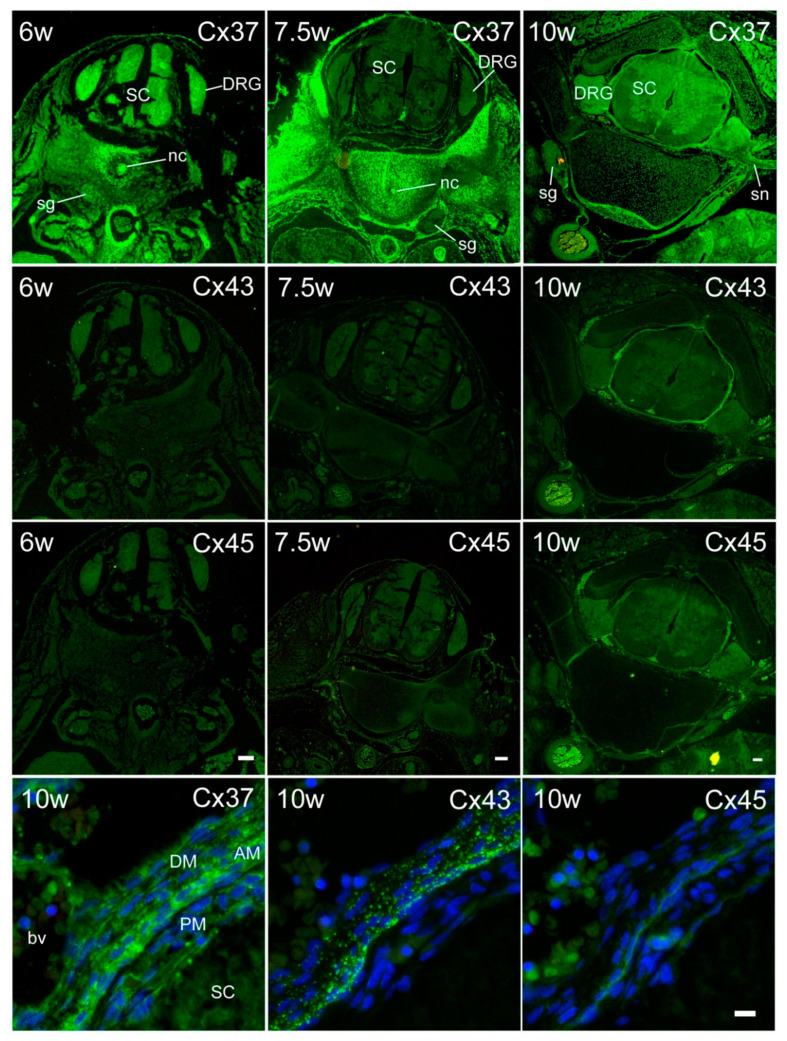
Expression of Cxs 37, 43, and 45 in the SC, ganglia, and meninges of human conceptuses. Thoracic segments of the SC of 6, 7.5, and 10 week-old human conceptuses were stained for Cx37, Cx43, and Cx45 (green). The strongest immunofluorescence was observed for Cx37 staining. SC—spinal cord; DRG—dorsal root ganglion; sg—sympathetic ganglion; sn—spinal nerve; nc—notochord. Objective magnification—4×; scale bars = 200 µm. In addition to the neural tissues, substantial Cx immunoreactivity was also observed in meninges (the lowest row). DM—dura mater; AM—arachnoid mater; PM—pia mater; bv—blood vessel. Objective magnification—40×; scale bar = 20 µm.

During early human development, we found expression of all three investigated Cxs, Cx37, Cx43, and Cx45, in all of the areas of interest in the SC (Figure 2). Results of semiquantitative analysis are presented in Supplementary Table S1. Moreover, a specific pattern of Cx expression was found in developing meninges (Figure 2). Cx37 was strongly immunoreactive in all three meningeal layers (pia mater—PM, arachnoid—AM, and dura mater—DM). However, the strongest immunofluorescence of Cx37 was observed in AM and the weakest in PM. Unlike Cx37, Cx43 immunofluorescence was not present in PM, but it was strong in AM and DM, with the strongest immunofluorescence found in AM. In contrast to Cx37 and Cx43, Cx45 immunoreactivity in meningeal layers was only weakly present in AM and PM, and it was absent from DM.

Expression of Cx37 seemed the highest in most of the structures analyzed, being significantly higher than the expression of Cx43 (*p* = 0.0087) and Cx45 (*p* = 0.032) in VIL (Figure 1). Although seemingly more Cx45 than Cx43 puncta were observed, no significant difference was found between the expression of Cx45 and Cx43.

The expression of Cx37 did not significantly differ between areas of interest themselves in the SC nor in comparison to the DRG. However, the expression of Cx43 was significantly higher in DRG when compared to the INL (*p* = 0.0212) and VIL (*p* = 0.0237), while the difference between DRG vs. DIL (when present) was *p* = 0.0521 and DRG vs. FP was *p* = 0.0505. In addition, the expression of Cx45 also did not significantly differ between areas of interest in the SC and the DRG.

We also compared the expression of different Cxs between distinct embryonic/early fetal periods: 5–6, 7–8, and 9–10 developmental weeks. We did not find significant differences in Cx37, Cx43, or Cx45 expression between the investigated periods in any of the areas of interest (when present). However, the tendency of the increase was observed for Cx37, which resulted in a significantly higher expression of Cx37 during 9–10 developmental weeks in comparison to Cx45 in VIL (*p* = 0.0417) and DRG (*p* = 0.0133) (Figure 1).

**Figure 3:**
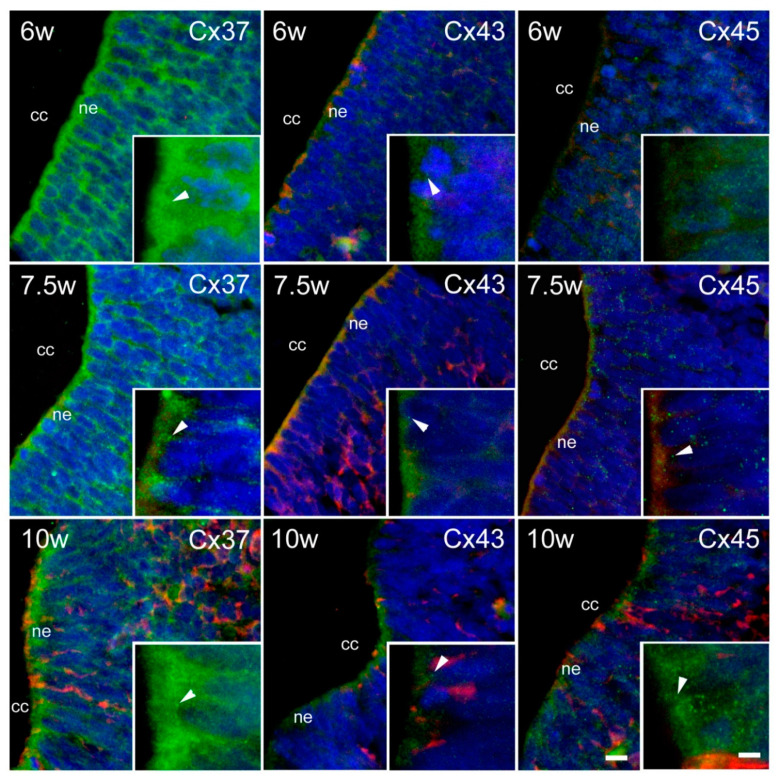
Expression of Cxs 37, 43, and 45 in the inner layer (INL) of the SC in human conceptuses. Thoracic segments of the SC of 6, 7.5, and 10 week-old human conceptuses were stained for Cx37, Cx43, and Cx45 (green), and protein gene peptide (PGP) 9.5 (red). The INL was presented on photomicrographs, with neuroepithelium (ne)—a layer in contact with the central canal (cc)—details shown in insets. Objective magnification—40× (scale bar = 20 µm); insets—100× (scale bar = 8 µm). Arrowheads are pointing to the granular pattern of Cx immunoreactivity in the cytoplasm of neuroepithelial cells.

**Figure 4:**
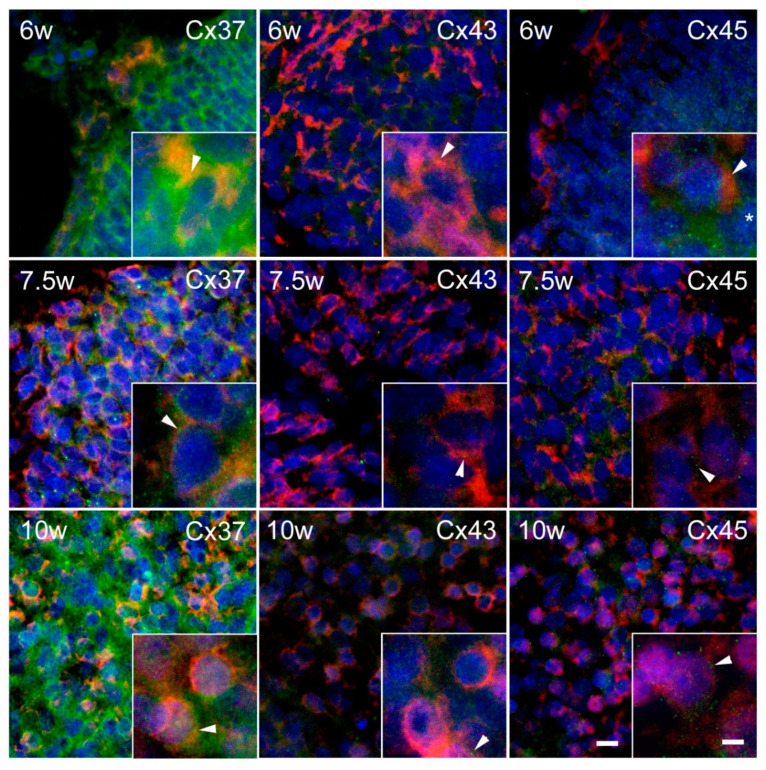
Expression of Cxs 37, 43, and 45 in the ventral part of the intermediate layer of the SC in human conceptuses. Thoracic segments of the SC of 6, 7.5, and 10 week-old human conceptuses were stained for Cx37, Cx43, and Cx45 (green), and PGP9.5 (red). The ventral part of the intermediate layer (VIL), corresponding to the basal plate, was presented on photomicrographs. Objective magnification—40× (scale bar = 20 µm); insets—100× (scale bar = 8 µm). Arrowheads are pointing to the yellow granular pattern of Cx/PGP9.5 co-localization in the cytoplasm of developing motor neurons. Asterisk—non-neuronal cell, presumably glia, immunoreactive for Cx45.

**Figure 5:**
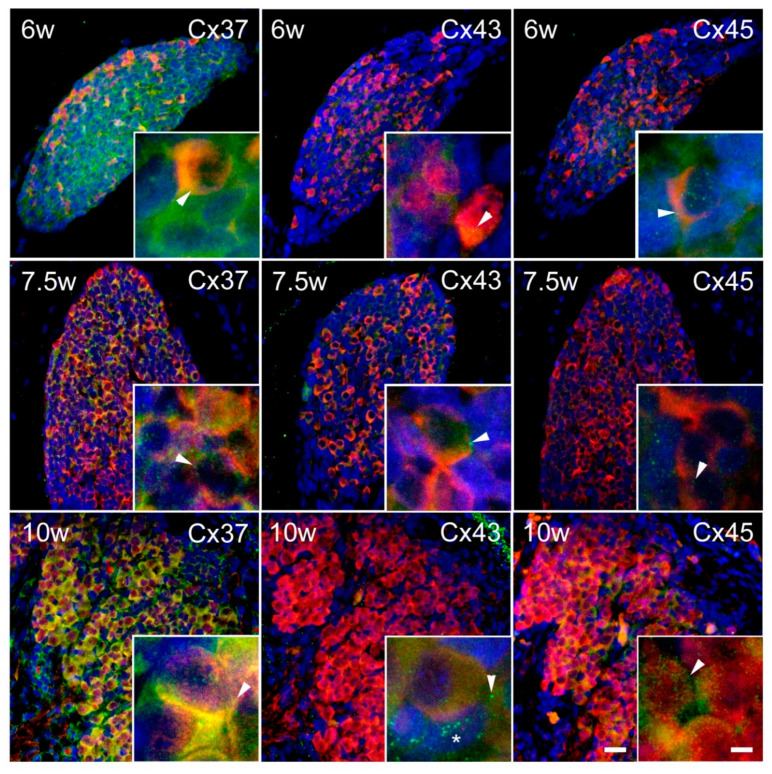
Expression of Cxs 37, 43, and 45 in the dorsal root ganglia (DRG) of the human conceptuses. Thoracic segments of the SC of 6, 7.5, and 10 week-old human conceptuses were stained for Cx37, Cx43, and Cx45 (green), and PGP9.5 (red).DRG are presented on photomicrographs. Objective magnification—10× (scale bar = 80 µm); insets—100× (scale bar = 8 µm). Arrowheads are pointing to the yellow granular pattern of Cx/PGP9.5 co-localization in the cytoplasm of developing primary sensory neurons. Asterisk—non-neuronal cell, presumably glia, immunoreactive for Cx43.

**Figure 6:**
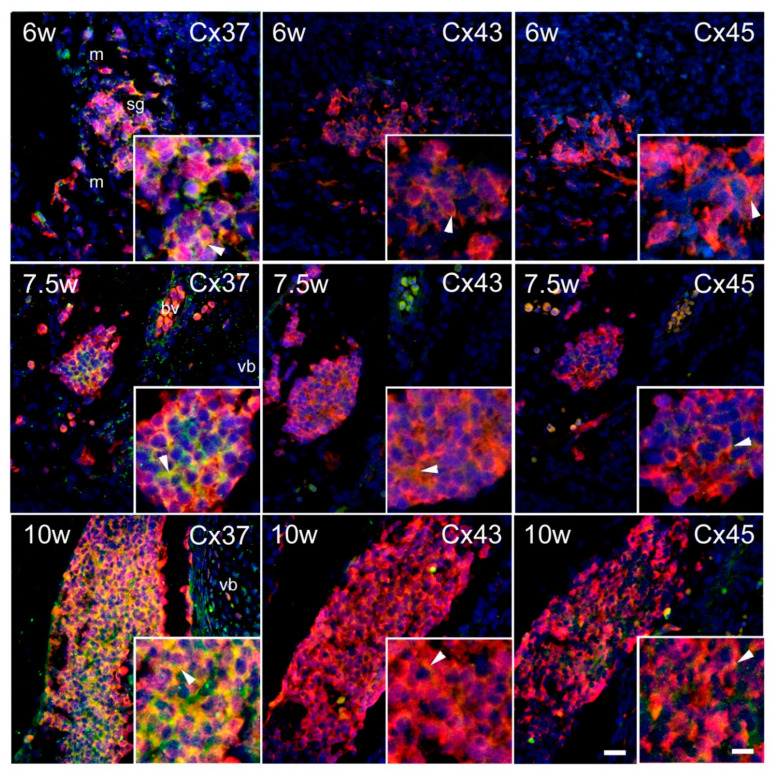
Expression of Cxs 37, 43, and 45 in the sympathetic (paravertebral) ganglia (sg) of the human conceptuses. Thoracic segments of the SC of 6, 7.5, and 10 week-old human conceptuses were stained for Cx37, Cx43, and Cx45 (green), and PGP9.5 (red). sg are presented on photomicrographs. Objective magnification—10× (scale bar = 80 µm); insets—40× (scale bar = 20 µm). Arrowheads are pointing to the yellow granular pattern of Cx/PGP9.5 co-localization in the cytoplasm of developing sympathetic neurons.

Substantial expression of Cxs was observed in the INL (Figure 3). In the intermediate zone of the SC (Figure 4), as well as in the DRG (Figure 5), persistent co-localization of all three types of Cxs with a protein gene product (PGP) 9.5, a marker for neurons, was found. However, it was evident that Cx expression was not restricted to the PGP9.5 immunoreactive neurons, since it was also present in PGP9.5 negative cells, including neuroepithelium of the INL (Figure 3), as well as the cells encircling neurons in DRG—presumably future satellite glia (Figure 5). In addition, expression of all three types of Cxs was found in developing ganglia of the sympathetic trunk (Figure 6), where it was similar to the expression found in DRG, also co-localizing with PGP9.5.

**Figure 7:**
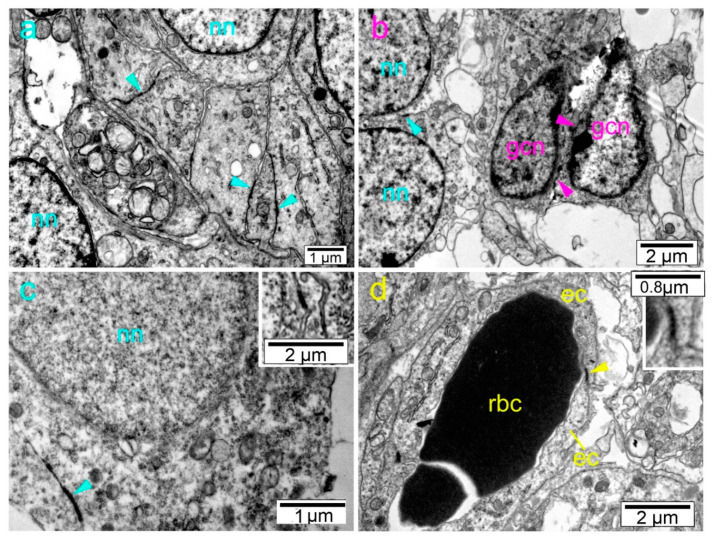
Transmission electron microscopy (TEM) photomicrographs of the developing human DRG. (**a**) nn—the nucleus of the neuron; gap junction between membranes of the neurons (blue arrowheads). (**b**) A gap junction between two neurons (blue arrowheads) and between two glial cells (magenta arrowheads). nn—neuronal nucleus; gcn—glial-cell nucleus. (**c**) Details of gap junction between two neurons (blue arrowheads) nn—neuronal nucleus. Inset: High magnification of the gap junctions between neurons. (**d**) A gap junction between two endothelial cells of the capillary (yellow arrowhead). Inset shows the marked detail. ec—endothelial cell; rbc—red blood cell in the capillary.

Transmission electron microscopy revealed the presence of numerous gap junctions between membranes of the neuronal cells and between neuronal cell processes. Gap junctions were also found between membranes of glial cells (Figure 7).

**Figure 8:**
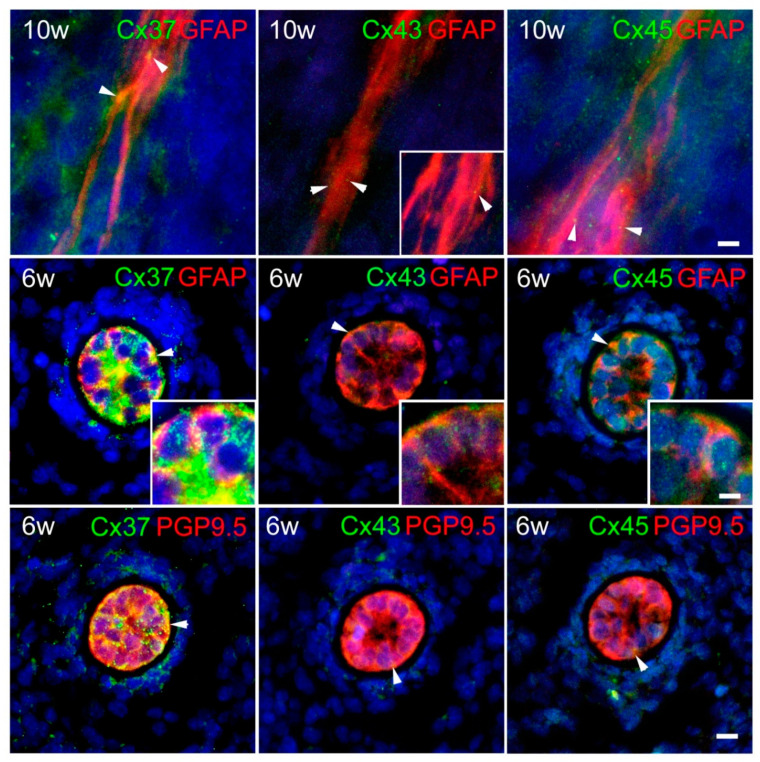
Co-localization of Cxs 37, 43, and 45 and glial fibrillary acidic protein (GFAP) and their expression in the notochord of the human conceptuses. Thoracic segments of the SC of 6 and 10 week-old human conceptuses were stained for Cx37, Cx43, and Cx45 (green), and glial fibrillary acidic protein (GFAP—red) or PGP9.5 (red). Co-localization of all three Cxs with GFAP was found in the roof plate of 10-week-old human fetus (first row). Strong expression of all three—Cx37, Cx43, and Cx45—were found in cells of notochord co-localizing with strong expression of GFAP (second row), as well as with even stronger immunoreactivity of PGP9.5 (third row). Objective magnification—100×, first row (scale bar = 8 µm for all); 40×—main figures on second and third row (scale bar = 20 µm); insets—100× (scale bar = 8 µm). Arrowheads are pointing to the yellow granular pattern of Cx/GFAP or PGP9.5 co-localization in the cytoplasm of the cells of the notochord.

In addition to co-localization with PGP9.5, we also found co-localization of Cx37, Cx43, and Cx45 with glial fibrillary acidic protein (GFAP) in the RP of a 10-week-old human fetus (Figure 8). Finally, we found a strong expression of all three—Cx37, Cx43, and Cx45—in the notochordal cells, where immunoreactivity of Cx37 was the strongest, while Cx43-immunoreactivity was the weakest. It is worth mentioning that the immunoreactivity of all three types of Cxs in the notochord was notably stronger in comparison to the immunoreactivity in the investigated neural tissues. Immunoreactivity of all three studied Cxs in the notochord persistently co-localized with a strong expression of GFAP, as well as with even stronger immunoreactivity of PGP9.5.

## 3. Discussion

Cxs are transmembrane proteins, building blocks of gap junctions, which in the SC are expressed in glial and neuronal cells. Gap junctions, as intercellular connections, allow direct electrical and metabolic communication between two adjacent cells, thus forming electrical synapses and coordinating different signal pathways [29], including ion transfer and propagation of the inflammation. It is also proposed that they have a role in the control of glial proliferation, myelination, and differentiation of neuronal stem cells [26,27,28]. In addition, releases of molecules such as potassium, glutamate, and ATP into the extracellular space via hemichannels under physiological conditions can modulate the neuronal activity or lead to induction of cell death in pathological conditions via autocrine or paracrine signaling [12,27,30]. All these substances, which can be exchanged through hemichannels, have a great influence on different signaling pathways. Therefore, Cxs channels are crucial for maintaining and coordinating the CNS activity [12] and mutations in genes, associated with individual isoforms of Cxs, can cause different pathological conditions of the nervous system [12,31,32,33]. More and more neurological diseases are associated with the disruption of Cxs and their channel functions in the CNS. There are three inherited diseases associated with Cx gene mutations. For example, Paznekas et al. found mutations in the Cx43 gene in patients with oculodentodigital dysplasia, autosomal dominant disorder [34]. Other diseases, based on functional modifications of various Cxs, are associated with a diverse spectrum of neurological diseases, from epilepsy and seizures to Alzheimer’s and motor neuron disease [35]. Furthermore, the connection between autism and changes in Cx43 expression was found [36]. During development, the neural GJIC (gap junctional intercellular communication) play a major role in coupling different cell types of the CNS and achieving a coordinated electrical transmission through electrical synapses [37]. Therefore, they have an impact on synaptic plasticity and influence on learning and memory [12]. Considering all the above-mentioned findings, spatio-temporal expression of Cxs during human embryonic development may be the basis in understanding how changes in a particular Cxexpression/function lead to pathological conditions.

During the SC development, Cxs are found in all cell types with different functional roles, ranging from cell migration and neuronal differentiation to forming neuronal activity patterns, which points to the relevance of specific spatio-temporal expression patterns for different Cx types [16,17]. Despite the extensive research of Cx’s role in the CNS development, there is still a gap in the knowledge about the expression of different types of Cxs in the embryonal and fetal development of the human SC. In this study, we aimed to characterize the expression of different types of Cxs in SC and DRG during early human developmental stages, which might imply their role in embryonal/early fetal SC development.

We found expression of all three investigated connexions, Cx37, Cx43, and Cx45, during early human development in all of the investigated areas of interest in the SC: neuroepithelium of the INL, the intermediate zone (DIL and VIL), RP, and FP. We also found extensive expression of all three Cxsin DRG and developing paravertebral ganglia of the sympathetic trunk. We compared the expression of different Cxs between distinct embryonic/early fetal periods: 5–6, 7–8, and 9–10 developmental weeks, but we did not find significant differences in Cx37, Cx43, or Cx45 expression between the investigated periods in any of the areas of interest.

Expression of Cxs in the neuroepithelium of the INL is congruent with multiple lines of evidence that have shown that Cxs have a major role in the regulation of neuronal progenitor cell proliferation as well as in differentiation and migration of neurons during the embryonic formation of the cerebral cortex. Namely, histological differentiation shows that neuroblasts or primitive nerve cells arise exclusively by the division of neuroepithelial cells [4,38]. In addition, Cxs signaling is involved in the reactivation of a latent stem cell niche after SC injury. Namely, Fabbiani et al. suggest that Cxs are involved in the early reaction of ependymal cells to injury, representing a potential target to improve the contribution of the central canal stem cell niche in the recovering of the tissue. Ependymal cells in the adult SC are latent progenitors that react to injury to support some degree of endogenous repair. Cx blockade reduced the injury-induced proliferation of ependymal cells [39]. Moreover, Russo and collaborators observed a high density of Cx43 on the end-feet of central canal-contacting cells in the turtle, which supported a key role of functional clustering via Cx43 in the SC neurogenesis [40].

In the postnatal hippocampus, neural progenitors are strongly coupled by gap junctions comprised of Cx43, and the deletion of Cx43 in vivo affects hippocampal neurogenesis [41]. Cx43 gap junctions are responsible for the hypothalamic tanycyte-coupled network. BrdU (bromodeoxyuridine) labelling demonstrated that the loss of Cx43 impairs hippocampal neurogenesis [42]. Furthermore, the virus-mediated ablation of Cxs in proliferative aNSC was also shown to reduce neurogenesis [43]. In the context of adult hippocampal neurogenesis, a double knockout model of Cx30 and Cx43 seemed to suggest that Cx30 and Cx43 affect neurogenic processes in an opposing manner, where Cx43 promotes survival of newborn neurons, and Cx30 restricts their survival [44,45].

Studies of the Cx expression in the development of human SC are rare. Zhan and Liu [46] did not find Cx43 protein expression in the posterior horn of the SC, but Cx43 was positive in the myelin sheath in the second to the third month of development. However, they detected Cx43 expression in some of the cells in the posterior horn of the SC in the fourth developmental month. Unlike their study, we found expression of Cx43, as well as Cx37 and Cx45, in developing sensory areas of the SC (AP) already in the sixth week of the embryonal development. Similar expression patterns we also found in the developing motor areas (BP). These findings agree with the results of the previous animal studies. Namely, electrical coupling between motor neurons in the CNS is a distinctive feature of neuronal development. Observing lumbar spinal motor neurons of neonatal rats, Chang et al. described the spatial and temporal patterns of motor neuron Cx expression. From embryonic life through adulthood, Cx36, Cx37, and Cx43 were evenly expressed, while expression of Cx40 and Cx45 decreased [47]. Chang et al. found strong expression of all studied Cxsin the ventral part of the developing rat SC, which is in agreement with our finding of Cx37, Cx43, and Cx45 in BP of the SC in human conceptuses in the similar phases of embryonic/early fetal development. The development of a neuroblast to a neuron in the ventral aspect of the SC includes an apolar and bipolar phase with primitive axon and dendrites formation. Finally, multipolar neuroblasts become adult nerve cells [4]. Cx overexpression was found to cause enhanced neurite outgrowth in PC12 cells treated with nerve growth factors to initiate neurogenesis [48]. Hence, the expression of Cx that we found in the developing motor (as well as the sensory) areas of the SC might be related to their function in neurite outgrowth.

In this study, we found predominant expression of Cx37 (over Cx43 and 45) in most of the SC structures analyzed, in the inner, as well as the intermediate layer. No significant difference was found between the expression of Cx45 and Cx43. The expression of Cx37 and Cx45 did not significantly differ between areas of interest in the SC or compared to the DRG. However, the expression of Cx43 was higher in DRG when compared to the INL, VIL, DIL, and FP. This specific spatial pattern of expression might reflect their relevance in SC and DRG development, with the highest relevance of Cx37 during differentiation of SC neurons and glia, and the approximately equal importance of all three studied Cxs during differentiation of DRG cells. Cx43 is the most extensively studied member of the Cx protein family during development. Jourdeuil and Taneyhill found Cx43 expression in the neural folds and pre-migratory neural crest cells during neural fold fusion and in migratory neural crest cells during epithelial-to-mesenchymal transition (EMT). All of the above can point to the key role of Cx43 in neural crest cells during EMT, migration, and gangliogenesis [49]. According to the present data, Cx37 expression is most prevalent in endothelial cells of the adult CNS [50,51]. During development, Cx37 plays a major role in vasculogenesis. In addition, the critical importance of Cx37 and Cx40 in maintaining endothelial communication for normal vascular development was found [52]. Endothelial cells in nerve tissue as a part of the blood–brain barrier (BBB) play a more complex role than anywhere else in the body, ensuring complete separation of circulating substances within the blood from the CNS parenchyma, thus providing a well-balanced microenvironment [53]. Therefore, a change in the amount of Cx37 causes various vascular abnormalities and could potentially affect the permeability status of the BBB [52,54].

During development, cells of sensory DRG originate from the neural crest cells and form two processes. The central processes, collectively known as the dorsal sensory root of the spinal nerve, penetrate the dorsal portion of the neural tube and after synapsing second neurons, ascend to the brain centers. The peripheral processes join fibers of the ventral motor roots and form a spinal nerve, terminating on sensory receptor organs [4]. Recent studies of neuropathic pain have also shown crucial roles of both Cx43 and pannexin-1 hemichannels in the SC and DRG neurons [55]. Our finding of strong Cx expression in DRG (as well as in the sympathetic ganglia) could be related to the already mentioned role of Cxs in the neurite outgrowth [48]. Also, it might have a role in the migration of the neural crest cells, which would have similarities with the role in the migration of the cortical neurons. Using shRNA (short hairpin RNA), Elias et al. achieved a significant reduction of Cx26 and Cx43 in rats and thus influenced the migration of neurons into the cortical plate and concluded that the migration was disrupted not because of altered gap junction channel functions, but because of their altered adhesion properties [14]. Cina et al. characterized the spatio-temporal expression of developmentally associated Cxs during the radial migration of newborn neurons to the cortical plate of the cerebral cortex. In the developing cortex, the expression of Cx26, Cx36, Cx37, Cx43, and Cx45 was present, while the expression of Cx30 and Cx32 were lacking and Cx40 was very low [13]. In another study, Cina et al. showed that neuronal migration will not occur if Cx43 is removed from the radial glia of mice, specifically if Cx43 lacks its cytoplasmic C-terminal domain [18]. Ruangvoravat and Lo [56] studied the expression of the Cx43 during mouse embryogenesis using in situ hybridization analysis from gestation days 4.5 to 12.5 and found that Cx43 transcripts are present in the neural tube of the 10.5-day embryo. Their results suggested that neural crest and sclerotome cells, i.e., cells that are presumably migratory, express high levels of Cx43 transcripts. Moreover, the finding of different Cxs in neural crest derivatives is in agreement with previous data about the importance of the gap junctions in the survival of spinal neural crest cells [57]. Besides the above-mentioned potential roles of Cxs, gap junctions play the role of electrical synapses that synchronize the activity of the neuronal cells in the developing and mature CNS, thus providing a supporting framework of neuronal activity before chemical synapses develop [58,59]. The amount of neuronal gap junctions is at its peak in the embryological and early postnatal periods, followed by a decline afterwards [15]. These data are in agreement with our TEM finding of neuron–neuron and neuron–glia connecting gap junctions in the developing human DRG.

In the intermediate zone of the SC, as well as in the DRG, persistent co-localization of all three types of Cxs with a PGP9.5, a marker for neurons, was found. However, it was evident that Cx expression was not restricted to the PGP9.5 immunoreactive neurons, but it was also present in PGP9.5 negative cells, including neuroepithelium of the INL, as well as the cells encircling neurons in DRG—presumably future satellite glia. The finding of Cxs in developing neurons of the SC might be related to the previously described role of Cx43 in neurogenesis in the brain [38]. In addition, it could also be related to the role of neuronal gap junctions in cell death caused by disturbed NMDA receptor function in developing neurons [60].

In addition to co-localization with PGP9.5, we also found co-localization of Cx37, Cx43, and C45 with GFAP in the RP of a 10-week-old human fetus. Coupling between glial cells enables the formation of glial syncytium, which has the role of Ca2+ waves propagation, metabolite transfers, and also an influence on extracellular concentration of K+ and thus on the activity of neurons [61]. Furthermore, the role of Cx43 in hemichannel formation and astrocyte migration appears especially relevant in brain injuries, since specific hemichannel inhibitory peptides abolish astrocyte migration, an important process required for glial scar formation [62]. In addition, it was found that astrocyte sigma-1 receptors modulate Cx43 expression, leading to the induction of mechanical allodynia in the SC injured mice [63]. Moreover, the data suggest that the astrocytic Cx43 hemichannels are negatively involved in the remyelination process by favoring local inflammation [64]. Decrock et al. described the importance of Cx channels in the interaction between neuronal, glial, and vascular compartments in the CNS. These channels allow fine regulation of different signaling pathways to ensure a proper response to a variety of information in order to maintain homeostasis in the CNS [12]. Hence, the finding of Cx co-localization with GFAP in developing SC might be related to the relevance of the glial Cxs during early fetal development in different, above-mentioned processes. Moreover, there is also a possibility that Cx43 and Cx45 may be assembled into heteromeric connexons and channels. Homomeric/homotypic gap junctions differ from heteromeric in conductivity, selectivity, and permeability [65]. Conduction, permeability, and gating properties of Cx43/Cx45 heteromeric gap junctions found in astrocytes [66] differ from homomeric channels of each individual Cx [67,68]. This could have an impact on the refinement of Cx-based intercellular communication during the nervous system development.

In this study, we found a specific pattern of Cx expression in developing meninges. Cx37 was strongly immunoreactive in all three meningeal layers (PM, AM and DM). However, the strongest immunofluorescence of Cx37 was observed in AM and the weakest in PM. Unlike Cx37, Cx43 immunofluorescence was not present in PM, but it was strong in AM and DM, with the strongest immunofluorescence found in AM. Opposite to Cx37 and Cx43, Cx45 immunoreactivity in meningeal layers was only weakly present in AM and PM, and it was absent from DM. These results point to the importance of cellular interconnectivity and synchronization in the development of human meninges.

Finally, we found a strong expression of all three, Cx37, Cx43, and Cx45, in cells of the notochord, where immunoreactivity of Cx37 appeared the strongest and Cx43-immunoreactivity the weakest. It is worth mentioning that the immunoreactivity of all three types of Cxs in the notochord was notably stronger in comparison to the immunoreactivity in the investigated neural tissues. Immunoreactivity of all three studied Cxs in the notochord persistently co-localized with a strong expression of GFAP, as well as with even stronger immunoreactivity of PGP9.5. As we already mentioned, the notochord provides a key signal (Shh), that induces neural cell differentiation of FP cells, motor neurons, and ventral interneurons [2,5,11]. We may assume that strong intercellular coupling of notochord cells might provide possible synchronization of these cells, which is important for regulation of signaling and establishment of morphogen gradient crucial for the specialization of ventral SC cell subtypes [5].

Concerning our results and the above-mentioned data from the literature, expression of Cx37, Cx43, and Cx45 in the SC, DRG, and sympathetic ganglia during early human development indicates their role in processes of neuronal formation, migration, and positioning in the developing human SC and ganglia. Hence, disturbances in their expression might lead to pathology of the nervous system development and result in malformations and neurological diseases. In addition to the already described direct role of Cxs in female infertility, by influence on the development of the germline, oogenesis, implantation, and decidualization [69,70], disturbance in Cx expression could potentially be a cause of the embryonal death due to malformation in the nervous system development.

## 4. Materials and Methods

### 4.1. Tissue Procurement and Processing

The human conceptuses were collected from the Department of Gynecology and Obstetrics and the Department of Pathology and processed with permission of the Ethical and Drug Committee of the University Hospital of Split in accordance with the Helsinki Declaration (class: 003-08/16-03/0001, approval number: 2181-198-03-04-16-0024; 1 August 2020) [71]. The poorly preserved material was discarded. The age of the conceptuses was estimated based on the external measurements (crown-rump length) and the Carnegie stages [72]. A total of 10 normal human conceptuses between 5 and 10 developmental weeks (Table 1) were collected from the University Hospital in Split, after spontaneous abortions or after tubal pregnancies. Conceptuses were dissected into 3 parts (cranial part, trunk, caudal part). Tissue was fixed in 4% paraformaldehyde in phosphate buffer saline, paraffin-embedded, cut in the transversal plane (5 µm), and mounted on glass slides.

### 4.2. Immunohistochemistry Procedure

The histological sections of thoracic segments were deparaffinized in xylene and rehydrated in water-ethanol solutions. Following deparaffinization and rehydration, the sections were heated in citrate buffer (pH 6.0) for 30 min in a steam cooker. After cooling to room temperature, the protein block solution (ab64226, Abcam, Cambridge, UK) was applied for 20 min. Then, the sections were incubated overnight with primary antibodies in a humid chamber (Table 2). After washing in phosphate buffer (PBS), appropriate secondary antibodies (Table 2) were applied for 1 h. For visualization of nuclei, DAPI (4′,6-diamidino-2-phenylindole) staining was used. Then, the slides were air-dried and cover-slipped (Immu-Mount, Shandon, Pittsburgh, PA, USA). The omission of the primary antibody from the procedure resulted in absence of staining of the tissue.

### 4.3. Transmission Electron Microscopy

For ultrastructural analysis, the samples of DRG were fixed in 4% paraformaldehyde in 0.1 M phosphate-buffered solution (pH 7.3) for 24 h, at 4 °C, and then post-fixed in 1% osmium tetroxide for 2 h. The samples were dehydrated in an ascending series of acetone and embedded in Durcopan resin (Fluka AG, Buchs, Switzerland) [73]. Ultrathin sections were cut from the chosen sites of interest at 0.050 µm and were counterstained with uranyl acetate and lead citrate [74]. The ultrathin sections were observed in a transmission electron microscope JEM JEOL 1010 (JEOL, Ltd., Tokio, Japan).

### 4.4. Data Acquisition and Analysis

Sections were viewed by immunofluorescence microscope (BX61, Olympus, Tokyo, Japan) and captured using a cooled digital camera (DP71, Olympus, Tokyo, Japan). The objectives used were: UPLFLN4X, UPLFLN10X2, UPLFLN40X, and UPLFLN100XO2 (all Olympus, Tokyo, Japan). In order to quantify Cx immuno-expression, visual fields captured at an objective magnification of 40× and constant exposure times were analyzed. Green granular deposits were interpreted as positive Cx37, Cx43, and Cx45 immuno-expression. Quantification of immunoreactivity was performed by using ImageJ (National Institutes of Health, Bethesda, MD, USA). Figures were prepared for analysis by using subtraction of the median filter 2 px and thresholded by using the color triangle threshold algorithm. Section percentage area covered by Cx immunofluorescence was measured in Adobe Photoshop, by manual outlining of the areas of interest. For the purpose of presentation, background subtraction and contrasting of microphotographs were conducted.

### 4.5. Statistical Analyses

GraphPad Prism 8.0.1 Software was used for statistical analyses (GraphPad Software, San Diego, CA, USA). N was 3 per group for 5–6 weeks and 7–8 weeks, and N was 5 for the 9–10 weeks group. Kruskal–Wallis test was used for comparison of the Cx expression in different developmental periods. A mixed model was used for repeated measures comparisons (comparison of different Cxs between themselves, and comparison of Cx expression in different parts of the SC/DRG). For comparison of different Cxs in a particular time-point (5–6, 7–8, and 9–10 weeks), the Friedman test was used due to the very small sample. Statistical significance was set up at *p* < 0.05.

## 5. Conclusions

Cxs as a part of the gap junctions have one of the key roles in regulating the cellular response to events in the environment, with fine coordination of nearby cells through the rapid exchange of ions and small molecules. The discovery of various CNS human diseases connected with the Cx expression indicates their great importance in tissue organization and cell communication. Hence, the spatio-temporal expression of Cxs during human development could help us in understanding why a deficiency or excess of individual Cxs leads to pathological conditions. In our study, spatio-temporal expression of Cx 37, 43, and 45 during the early human developmental stages was characterized, thus supporting their possible role in processes of neuronal formation, migration, and positioning in the developing human SC and ganglia.

## Figures and Tables

**Table 1:** The human conceptuses analyzed in this study.

Age (Weeks)	Crown-Rump Length (CRL) (mm)	Carnegie Stage	No.
5	8	14	1
6	14	16	2
7	21	20	2
8	27	22	1
9	32	-	2
10	36	-	2

**Table 2:** Primary and secondary antibodies.

Primary	**Antibody**	**Code no.**	**Host**	**Dilution**	**Source**
	Anti-Cx37/GJA4	ab181701	Rabbit	1:100	Abcam, Cambridge, UK
	Anti-Connexin 43/GJA1	ab87645	Goat	1:300	
	Anti-Connexin 45/GJA7/Cx45	ab135474	Rabbit	1:100	
	Anti-GFAP	ab53554	Goat	1:100	
	Anti-GFAP (2E1)	sc-33673	Mouse	1:50	Santa Cruz Biotechnology Inc., Dallas, TX, USA
	PGP9.5 Monoclonal Antibody (BH7)	480012	Mouse	1:500	Invitrogen
Secondary	AlexaFluor^®^488 Donkey Anti-Rabbit IgG	711-545-152	Donkey	1:400	Jackson Immuno Research Laboratories Inc., Baltimore, PA, USA
	Rhodamine Red™-X (RRX) Donkey Anti-Goat IgG	705-295-003			
	Rhodamine Red™-X (RRX) Donkey Anti-Mouse IgG	715-295-151			
	AlexaFluor^®^488 Donkey Anti-Goat IgG	705-545-003			

## Supplementary Materials

The following are available online at https://www.mdpi.com/1422-0067/21/24/9356/s1.

## Abbreviations

SC	spinal cord
DRG	dorsal root ganglia
Cx	connexin
CNS	central nervous system
Shh	Sonic hedgehog
BMPs	bone morphogenetic proteins
INL	inner layer
DIL	dorsal part of the intermediate layer
VIL	ventral part of the intermediate layer
RP	roof plate
FP	floor plate
PGP9.5	protein gene product 9.5
PM	pia mater
AM	arachnoid
DM	dura mater
GFAP	glial fibrillary acidic protein
ATP	adenosine triphosphate
AP	alar plate
BP	basal plate
TEM	transmission electron microscopy
PBS	phosphate buffer saline
sg	sympathetic ganglion
