# Supplementary material for: Expression of Connexins 37, 43 and 45 in Developing Human Spinal Cord and Ganglia
Source: Int J Mol Sci. 2020 Dec 8;21(24):9356. doi: 10.3390/ijms21249356 (PMC7770599; doi:10.3390/ijms21249356)
Supplement: Supplementary file 1 [file ijms-21-09356-s001.pdf]

International Journal of
Molecular Sciences

Supplementary Table S1. Expression of different connexins in areas of interest—semiquantitative analysis.

	**Cx37**			**Cx43**			**Cx45**		
	**5–6 weeks**	**7–8 weeks**	**9–10 weeks**	**5–6 weeks**	**7–8 weeks**	**9–10 weeks**	**5–6 weeks**	**7–8 weeks**	**9–10 weeks**
**INL**	++	++	+++	+	+	+	+	++	+
**DIL**	++	++	+++	+	+	+	+	+	+
**VIL**	++	++	+++	+	+	+	+	+	+
**DRG**	+++	++++	++++	++	++	++	+	+	++
**sg**	++	+++	+++	+	+	++	+	+	++
**nc**	++++	++++	+++	++	++	++	++	++	++

INL—inner layer; DIL—dorsal intermediate zone; VIL—ventral intermediate zone; DRG—dorsal root ganglion; sg—sympathetic ganglion; nc—notochord.
